# Designer Lipid-Like Peptides: A Class of Detergents for Studying Functional Olfactory Receptors Using Commercial Cell-Free Systems

**DOI:** 10.1371/journal.pone.0025067

**Published:** 2011-11-23

**Authors:** Karolina Corin, Philipp Baaske, Deepali B. Ravel, Junyao Song, Emily Brown, Xiaoqiang Wang, Christoph J. Wienken, Moran Jerabek-Willemsen, Stefan Duhr, Yuan Luo, Dieter Braun, Shuguang Zhang

**Affiliations:** 1 Center for Biomedical Engineering, Massachusetts Institute of Technology, Cambridge, Massachusetts, United States of America; 2 NanoTemper Technologies GmbH, München, Germany; 3 Systems Biophysics, Functional Nanosystems, Ludwig-Maximilians University Munich, München, Germany; 4 Center for Bioengineering and Biotechnology, China University of Petroleum (East China), Qingdao, Shandong, China; 5 Department of Pharmaceutical Sciences, School of Pharmacy, University of Maryland, Baltimore, Maryland, United States of America; University of Milan-Bicocca, Italy

## Abstract

A crucial bottleneck in membrane protein studies, particularly G-protein coupled receptors, is the notorious difficulty of finding an optimal detergent that can solubilize them and maintain their stability and function. Here we report rapid production of 12 unique mammalian olfactory receptors using short designer lipid-like peptides as detergents. The peptides were able to solubilize and stabilize each receptor. Circular dichroism showed that the purified olfactory receptors had alpha-helical secondary structures. Microscale thermophoresis suggested that the receptors were functional and bound their odorants. Blot intensity measurements indicated that milligram quantities of each olfactory receptor could be produced with at least one peptide detergent. The peptide detergents' capability was comparable to that of the detergent Brij-35. The ability of 10 peptide detergents to functionally solubilize 12 olfactory receptors demonstrates their usefulness as a new class of detergents for olfactory receptors, and possibly other G-protein coupled receptors and membrane proteins.

## Introduction

Olfactory receptors are arguably the most sensitive detectors: they distinguish between thousands of odorants down to parts per billion or trillion. Although they comprise the largest class of receptors, no molecular structure currently exists, and the molecular basis of olfaction remains an enigma. As members of the GPCR family, olfactory receptors have 7-transmembrane regions that make them unstable outside of their native lipid bilayer. It is thus necessary to find an optimal detergent that is capable of keeping them soluble, stable, and functional.

Although selecting an appropriate detergent is crucial for membrane protein studies, it is a daunting task. A bewilderingly large selection of detergents is available, and the optimal detergent for a protein must be empirically determined [1]. To complicate matters, detergents that are optimal for one application may not be appropriate for others. For example, detergents that best solubilize proteins from cell membranes often cause destabilization or denaturation in the long run. Additionally, detergents appropriate for biochemical assays may inhibit protein crystallization [1], [2]. Careful screening is necessary, but is a time consuming and expensive process. Finding an appropriate detergent has thus become the critical bottleneck not only for olfactory receptors and other membrane protein studies, but also for designing and producing membrane proteins for biotechnological devices.

The limitations and problems of using traditional detergents highlight the need for a general class of detergents that can work with diverse membrane proteins. Several attempts have been made, including the design of amphipathic helical peptides, lipopeptides, amphipols, and tripod amphiphiles [3]–[8]. However, these detergents are expensive, difficult to manufacture, or heterogeneous. Also, some cannot be used with many proteins, or cannot maintain proteins soluble and functional for sufficient periods of time.

We previously reported a class of peptide detergents designed to behave like common detergents. These peptide detergents had defined critical aggregation concentrations (CAC), and formed nanostructures including micelles, nanovesicles and nanotubes [9]–[13]. They also interacted well with lipids to form monoolein bilayers [14]. We further showed that they could solubilize and stabilize diverse multi-transmembrane proteins, including Glycerol-3-phosphate dehydrogenase [15], photosystem I [16], [17], and a handful of G-protein coupled receptors (GPCRs) [18], [19].

**Figure 1:**
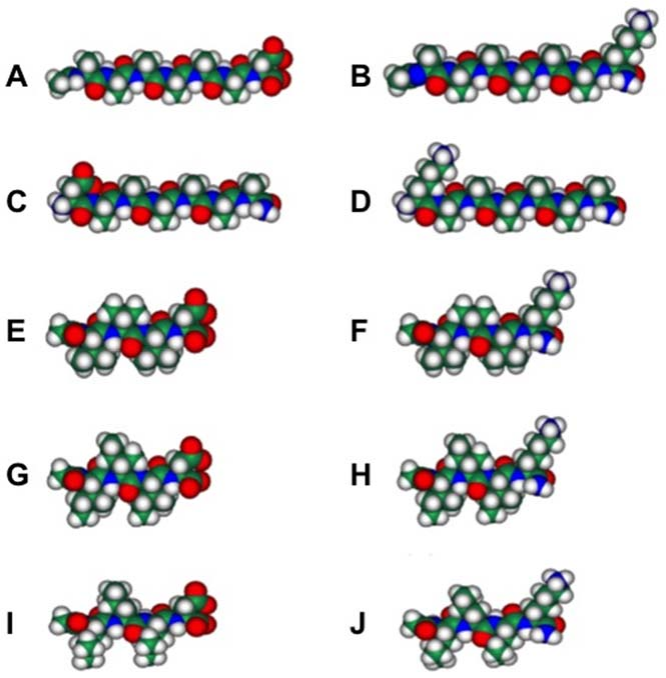
Molecular models of peptide detergents at neutral pH. A) Ac-AAAAAAD-COOH. B) Ac-AAAAAAK-CONH_2_. C) DAAAAAA-CONH_2_. D) KAAAAAA-CONH_2_. E) Ac-VVVD-COOH. F) Ac-VVVK-CONH_2_. G) Ac-IIID-COOH. H) Ac-IIIK-CONH_2_. I) Ac-LLLD-COOH. J) Ac-LLLK-CONH_2_. Aspartic acid (D) is negatively charged and lysine (K) is positively charged. The hydrophobic tails of the peptide detergents consist of alanine (A), valine (V), isoleucine (I) and leucine (L). Each peptide is ∼2–2.5 nm long, similar size to biological phospholipids. Color code: teal, carbon; red, oxygen; blue, nitrogen and white, hydrogen.

Here we report the use of short designer lipid-like peptide detergents (Figure 1) to functionally solubilize 12 unique olfactory receptors. The peptide detergents' performance was comparable to Brij-35, a common detergent. The ability of the tested peptide detergents to solubilize a large number of olfactory receptors equally as well as the best detergent demonstrates their potential as a class of detergents for olfactory receptors and perhaps other membrane protein studies.

## Results

### Systematic Detergent Screening

**Figure 2:**
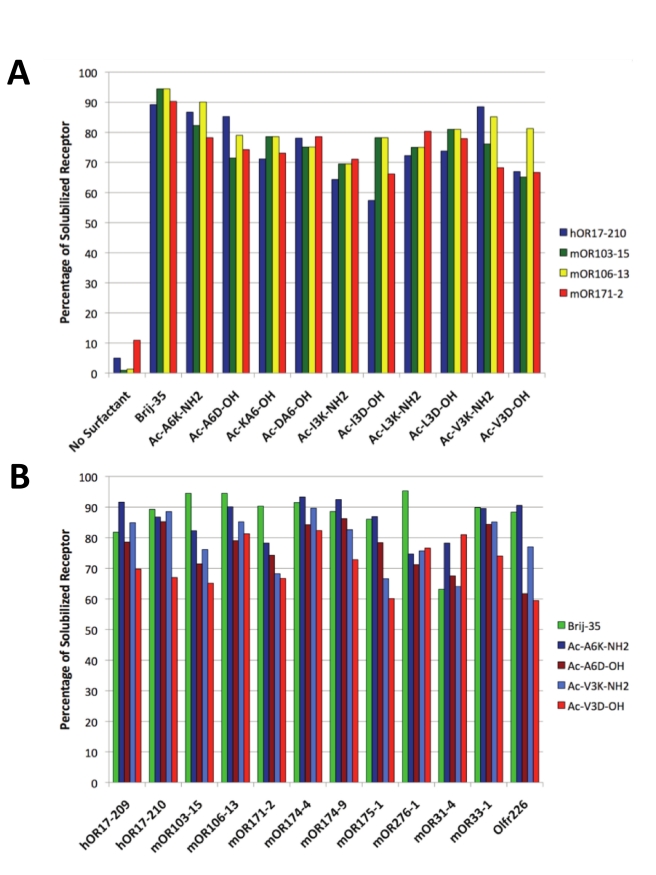
Olfactory receptor solubility in Brij-35 and peptide detergents. Each receptor was expressed in the presence of Brij-35 or a peptide detergent using a commercial *E.coli* cell-free expression system. Upon completion of the reactions, the samples were centrifuged to separate solubilized receptor from insoluble aggregates. The soluble fraction was removed, and the pellet was resuspended in an equal volume of buffer. Soluble and precipitated protein samples were analyzed with Western and dot blots using the rho1D4 monoclonal antibody; relative sample intensities were used to calculate the percentage of solubilized receptor. As controls, reactions with no peptide or detergent were assayed. A) The presence of a detergent was necessary to solubilize the olfactory receptors, and all of the peptide detergents were able to solubilize four unique receptors. B) The detergent peptides and Brij-35 were able to solubilize similar fractions of protein. Peptides that were positively charged or had longer tails tended to solubilize higher fractions of receptors.

Systematic screens were performed to assess the ability of peptide detergents to produce and solubilize 12 olfactory receptors in a commercial *E.coli* cell-free expression system. First, the ability of diverse peptides to function as detergents was tested. Four olfactory receptors were selected and produced in the cell-free system in the presence of all 10 peptides. The soluble and insoluble protein fractions were compared (Figure 2A). Second, the ability of peptides to solubilize a wide variety of olfactory receptors was tested by comparing the solubility of all 12 olfactory receptors in 4 peptide detergents and Brij-35 (Figure 2B). A detergent screen showed that Brij-35 was the optimal traditional detergent for producing olfactory receptors in the cell-free system [20]. Brij-35 was thus used as a control: for each test mentioned above, the peptide detergents' performance was compared to that of Brij-35. Reactions with no detergent served as additional controls.

Western and dot blot analysis was used to compare the soluble and insoluble receptor fractions. Figure 2A shows that a detergent is necessary to solubilize the olfactory receptors. Without detergent, only ∼10% of the produced receptor is soluble. With a proper detergent, up to 95% of the expressed receptor remains soluble. The peptides and Brij-35 maintained similar fractions of olfactory receptors soluble. Brij-35 solubilized 63–95% of the expressed olfactory receptors, with most receptors having soluble fractions between 80–90%. The peptide detergents solubilized 57–93% of the expressed olfactory receptors, with most olfactory receptors having fractions between 75–90%. Figure 2B shows that 4 peptide detergents solubilized ∼60–90% of all 12 olfactory receptors, suggesting their potential use as a general class of detergents for additional olfactory receptors and perhaps a wider range of GPCRs and other membrane proteins.

### Determination of Receptor Yields

**Figure 3:**
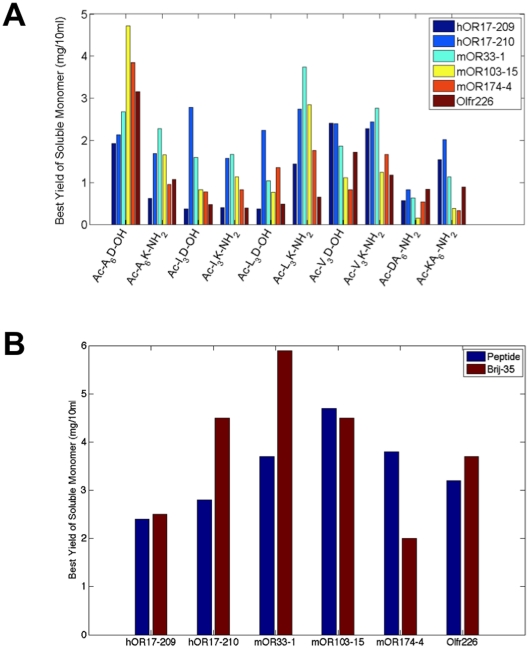
Detergent peptides can yield milligram quantities of solubilized olfactory receptors. The total receptor yield is dependent on the peptide used. A) The maximum expected yields of solubilized monomer for 6 of the receptors in the presence of each peptide. To determine the expected yields, solubilized receptor and protein with a known concentration were compared on a Western blot. The relative intensities of the known protein sample and the test samples were used to calculate the maximum receptor yields. B) The maximum yield of the monomeric form of all tested olfactory receptors expected in a 10 ml reaction. Only results from the most effective detergent peptide are shown. The total protein yield is dependent on the peptide detergent used. These results are compared to the yields of receptors made in Brij-35. In most cases, the yields are comparable. For two receptors, Brij-35 resulted in more expressed protein, and for one receptor a peptide resulted in more. The maximum yields for hOR17-210 and mOR103-15 are the same as those previously reported [19], [20].

The maximum yields of olfactory receptors produced in the presence of the peptide detergents were estimated by comparing their band intensity to that of a receptor with a known concentration. Milligram quantities of receptor could be produced, demonstrating that cell-free synthesis in the presence of peptide detergents is a good alternative for large-scale olfactory receptor production, and perhaps for others GPCRs as well. However, as with the solubilization results, the protein yield depended on both the specific receptor and the specific peptide detergent used (Figure 3A). At least 2 mg of hOR17-210 and mOR33-1 could be produced in a 10 ml *E.coli* cell-free reaction with at least 5 of the peptide detergents. The peptide Ac-A_6_D-OH yielded the largest amount: ∼4.8 mg of mOR103-15 per 10 ml reactions. Six other peptides were able to produce at least ∼2.5 mg for at least one olfactory receptor: Ac-A_6_K-NH_2_, Ac-I_3_D-OH, Ac-L_3_D-OH, Ac-L_3_K-NH_2_, Ac-V_3_D-OH, and Ac-V_3_K-NH_2_. Overall, at least 2 mg of most receptors could be synthesized in a 10 ml reaction in the presence of at least one of the peptide detergents. These amounts were comparable to those that could be obtained using Brij-35 (Figure 3B), as well as those that could be obtained in a smaller-scale study [19], .

### Olfactory Receptor Purification and Purity Analysis

**Figure 4:**
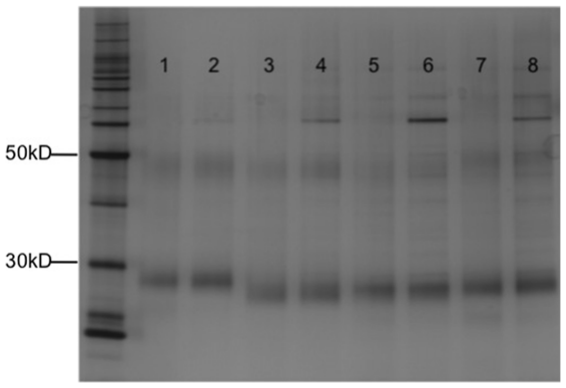
Silver stain of four purified olfactory receptors produced in either Brij-35 or a peptide. Lane 1: mOR103-15 made in Brij-35. Lane 2: mOR103-15 made in Ac-V_3_D-OH. Lane 3: mOR174-4 made in Brij-35. Lane 4: mOR174-4 made in Ac- V_3_D -OH. Lane 5: mOR174-9 made in Brij-35. Lane 6: mOR174-9 made in Ac-A_6_D-OH. Lane 7: Olfr226 made in Brij-35. Lane 8: Olfr226 made in Ac-A_6_K-NH_2_. The Brij-35 and peptide produced samples have similar purities. Running at the same length and showing the same tendency to dimerize indicates that the peptides do not interfere with full-length protein translation, or proper receptor folding or function.

Four olfactory receptors were selected for larger scale expression and purification for structural and functional analysis. The olfactory receptors mOR103-15, mOR174-4, mOR174-9, and Olfr226 were expressed using Brij-35 or a peptide detergent, and purified using the rho1D4 monoclonal antibody. The purifications were performed in the presence of fos-choline 14 (FC14) because it has been shown to be the preferred detergent for olfactory receptor purification [21]–[23], and because a common buffer for the Brij-35 and peptide-produced receptors allows for direct comparison between the two samples. Moreover, a detergent exchange was necessary in order to carry out subsequent analyses, as the peptides elicit signals that are difficult to distinguish from the receptor signals. Because a simple detergent exchange is unlikely to re-fold misfolded receptors, the structures of the olfactory receptors initially produced in Brij-35 or a peptide should not be positively affected by the change to FC14. The purified receptors were analyzed on a western blot and silver stain (Figure 4). The silver stain shows that receptors expressed in Brij-35 or a peptide could be purified up to >80% purity using immunoaffinity chromatography alone. Olfactory receptors produced in both the Brij-35 and peptide detergent were the same size, and exhibited the expected monomeric and dimeric bands. These results suggest that the peptide detergents do not interfere with full-length cell-free protein expression. They further suggest that the peptide detergents do not interfere with proper receptor folding or structure, as the expressed proteins show the tendency to dimerize.

### Secondary Structure Analysis Using Circular Dichroism

Circular dichroism (CD) was used to assess the secondary structure of the 4 purified olfactory receptors. The purification and CD analyses of olfactory receptors were performed in FC-14 because the peptides themselves have strong CD signals that interfere with and overwhelm the receptor signals.

**Figure 5:**
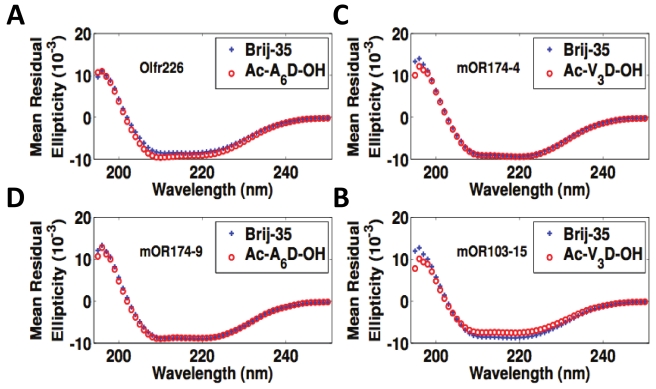
CD spectra of Brij-35 and peptide detergent-produced olfactory receptors. A) Olfr226, B) mOR174-4, C) mOR174-9, and D) mOR103-15. All eight samples have characteristic secondary alpha-helical structures, suggesting that the receptors are properly folded. The near overlap of the peptide and detergent curves indicates that the peptides function as detergents equally as well as traditional detergents. The spectra for mOR103-15 are the same as those previously reported [19], [20], and are shown together here for comparison.

Figure 5 directly compares the CD spectra of olfactory receptors produced in either Brij-35 or a peptide detergent. Figure 5A shows Olfr226 produced in Brij-35 and Ac-A_6_K-NH_2_; Figure 5B shows mOR174-4 produced in Brij-35 and Ac-V_3_D-OH; Figure 5C shows mOR174-9 produced in Brij-35 and Ac-A_6_D-OH; Figure 5D shows mOR103-15 produced in Brij-35 and Ac-V_3_D-OH. All purified receptors have characteristic α-helical spectra, with signature valleys at 220 nm and 208 nm. Because GPCRs have 7-transmembrane alpha-helical domains, these CD spectra indicate that these olfactory receptors are properly folded. Moreover, the nearly superimposed spectra for olfactory receptors produced in Brij-35 or peptide detergent indicate that the peptide detergents are able to structurally stabilize these olfactory receptors equally as well as traditional detergent Brij-35.

### Ligand Binding Analysis Using Microscale Thermophoresis

Microscale thermophoresis was used to determine whether the expressed and purified proteins were functional. This approach is based on the ligand binding-induced change in movement of molecules along a temperature gradient [24], [25], and is capable of detecting interactions with ligands as small as calcium ions [26]. Because olfactory receptors are larger than 35 kDa and their ligands are smaller than 300 Da, it was necessary to use thermophoresis to detect ligand binding instead of less sensitive methods like SPR or quartz crystal microbalance. All 8 receptors used with CD were analyzed with their known odorants (Table 1). Boiled receptors were used as controls.

**Table 1:** Olfactory receptor Ligands and Measured EC50 Values.

Receptor	Surfactant Used	Ligand	Measured EC50 (µM)
mOR103-15	Brij-35	Heptanal	2±0.7
mOR103-15	Ac-V_3_D-OH	Heptanal	0.9±0.2*
mOR174-4	Brij-35	Ethyl Vanillin	7±2
mOR174-4	Ac-V_3_D-OH	Ethyl Vanillin	4±2
mOR174-9	Brij-35	Ethyl Vanillin	4.9±3.5
mOR174-9	Ac-A_6_D-OH	Ethyl Vanillin	5.1±2
Olfr226	Brij-35	2,4-DNT	86±36
Olfr226	Ac-A_6_K-NH_2_	2,4-DNT	3±1.6

*This measurement was obtained at a longer time (25 s vs. 15 s) and lower IR-laser power (1.5 V v. 2.5 V) than the other measurements. The constant for mOR103-15 made in Ac-V_3_D-OH is the same as that reported in [19], and is shown here for comparison with the same receptor made in Brij-35.

**Figure 6:**
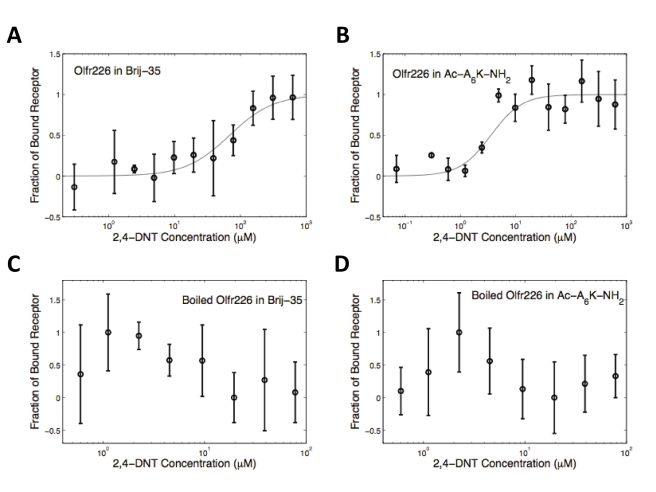
Ligand binding of olfactory receptor Olfr226 to its ligand 2,4-DNT: A) Olfr226 produced in Brij-35, B) Olfr226 produced in Ac-A_6_K-NH_2_ C) Olfr226 produced in Brij-35 and boiled, and D) Olfr226 produced in Ac-A_6_K-NH_2_and boiled. Receptors produced in Brij-35 or a peptide exhibit typical sigmoidal binding curves. There is one plateau at low concentrations and another at high concentrations, while the boiled controls have no plateaus. This suggests that the thermophoresis signals are measuring ligand binding. All curves were normalized to the fraction of bound receptor. Open circles show the mean measurements from 3 experiments; the lines through the points are the best-fit curves using the Hill equation. The binding results shown here are representative of the data from all four OR samples.

Figure 6 and Table 1 show the ligand-binding results of the Brij-35- and peptide detergent-solubilized olfactory receptors, and their boiled controls. All of the receptors exhibited a typical sigmoidal binding curve. In contrast, the negative controls had random amplitudes throughout the odorant titration range. These results indicate that all of the olfactory receptors bound their respective odorants. The large noise in the boiled controls is probably due to the presence of protein aggregates of different size, and hence different diffusive and thermophoretic properties. Olfr226 produced in peptide detergent exhibited a significantly higher affinity for its ligand than receptor produced in Brij-35. The other olfactory receptors had similar binding affinities in both types of detergents. These results show that both classes of detergents are able to aid in the production and solubilization of functional olfactory receptors, and that peptides may confer more functional stability to some solubilized receptors. The measured EC_50_ value for each tested olfactory receptor is in the micromolar range, which is consistent with previous reports [22], [23].

## Discussion

This study showed that short peptide detergents solubilized and functionally stabilized cell-free produced olfactory receptors equally as well as the detergent Brij-35. All of the tested peptide detergents were able to solubilize all of the tested olfactory receptors. Soluble olfactory receptor fractions were as high as 93%. We previously reported lower solubilities for hOR17-210 and mOR103-15 in the presence of some peptides [19]. This difference in solubility is probably due to differences in the peptide batches, or the dynamic nature of the peptides. Previous reports have noted the peptides' ability to form various mesoscale structures, and to change between structures over time [10]. It is probable that specific structures are better able to solubilize the expressed receptors, and more research needs to be done to elucidate these effects. However, the comparable results between the peptides and Brij-35 for 12 olfactory receptors reported here demonstrate their potential to be used as detergents for membrane protein studies.

The results in our study show that the efficacy of solubilization primarily depends on the peptide detergent properties. Figure 2B shows that the cationic peptides usually solubilized a greater fraction of expressed protein than their anionic counterparts. This effect was more pronounced for the longer peptides (12 out of 12 olfactory receptors with Ac-A_6_K-NH_2_ and Ac-A_6_D-OH) than for the shorter peptides (10 out of 12 olfactory receptors with Ac-V_3_K-NH_2_ and Ac-V_3_D-OH). Figure 2A indicates that this effect may depend on the specific receptor. The receptors hOR17-210 and mOR171-2 were consistently more soluble in cationic peptides, while the receptors mOR103-15 and mOR106-13 were often more soluble in anionic peptides. Because mOR103-15 and mOR106-13 had greater solubility in the more hydrophobic anionic peptides (leucine and isoleucine tails), it is probable that the tail composition can alter tendencies caused by the head group properties. Additionally, peptides with longer hydrophobic tails typically had higher soluble fractions than those with the same charge but shorter tails (10 receptors for the cationic peptides, 9 for the anionic) (Fig. 2B). Figure 3A further suggests that subtle changes in the residue order may affect how the peptides interact with the olfactory receptors expression. Although composed of the same amino acids, Ac-A_6_D-OH yielded significantly more protein than Ac-DA_6_-NH_2_. A similar result was observed with most tested olfactory receptors with Ac-A_6_K- NH_2_and Ac-KA_6_- NH_2_. These results further indicate the peptide charge or ionic character can greatly affect olfactory receptor solubility or expression. Additional experiments will be needed to fully characterize and understand the effects of peptide properties on olfactory receptor solubilization and production.

CD and microscale thermophoresis demonstrated that peptide- and Brij-35- produced olfactory receptors had similar structures and binding affinities. All of the purified receptors had characteristic α-helical spectra, which is expected for the 7-transmembrane helix members of the GPCR family. Moreover, the spectra of the Brij-35 and peptide-purified olfactory receptors nearly overlapped, indicating that the peptides were able to aid in the proper folding of expressed olfactory receptors equally as well as Brij-35. The peptide-produced proteins had similar or higher-affinity binding constants as the Brij-35-produced proteins. This further underscores the potential usefulness of peptides as detergents in membrane protein studies. Indeed, the greater stability implied by the tighter binding suggests that the peptides may even be able to facilitate membrane protein crystallization. Further studies are necessary to determine the lifetime of a functional receptor in a peptide detergent. However, the results reported here, as well as previous studies [15]–[19], indicate that that the peptides may be able to fulfill just such a function, perhaps better than many traditional detergents. Furthermore, our findings are particularly important because they suggest that the peptides are a general class of detergents that can be used with cell-free expression methods. Although cell-free production is a mature technology for soluble proteins [27]–[30], few membrane proteins have been produced, and even then only through laborious detergent screens [23], [31]–[33]. The methods reported here may help accelerate the production of many more membrane proteins for structural studies and biotechnological advancements.

The peptides detergents described here may be useful for diverse membrane protein studies. They function comparably to traditional detergents, and offer several advantages over other novel detergents. Their properties are similar to commonly used detergents, they can be systematically designed and economically produced at high purity, and they remain stable for long periods of time. The ability of every tested peptide to solubilize 12 olfactory receptors from three different species, and of 3 peptide detergents to functionally stabilize 4 olfactory receptors further suggests that they may be a general class of detergents capable of functionally solubilizing a wider range membrane proteins.

Further studies are needed to characterize detergent peptide property effects on membrane proteins; it may be possible to rationally design a detergent optimal for a given protein. Future studies are also needed to analyze the long-term stability of membrane proteins solubilized in the peptide detergents. However, our current study suggests that peptide detergents are promising for membrane protein studies, and could not only lead to a better understanding of olfactory receptors and other GPCRs, but they could also be used in the design and fabrication of olfactory receptor-based biotechnological devices.

## Materials and Methods

### Peptide Design and Synthesis

Cationic, anionic, and zwitterionic peptide detergents were designed. Lysine or aspartic acid was used for the hydrophilic head. To control the detergent ionic nature, each peptide was capped by acetylation at the N-terminus, amidation at the C-terminus, or both. Alanine, valine, leucine, and isoleucine were used for the hydrophobic tails, which were either 3 or 6 residues in length. The traditional detergent Brij-35 was used as a control because it was experimentally determined to be the optimal detergent for most of the tested receptors. Molecular models of the peptide detergents are shown in Figure 1.

All peptides were synthesized and purified by CPC Scientific Inc., CA. The peptides, received in powder form, were dissolved in milli-Q water, sonicated, and adjusted to a pH value above 7.0 with NaOH or HCl to increase peptide solubility. The suspension was then filtered through a 0.22 µm filter to remove insoluble particles and stored at room temperature.

### Olfactory Receptor Design

Protein sequences of 12 olfactory receptors were obtained from the NCBI online database: hOR17-209 (NP_003546.1), hOR17-210 (SwissProt Q8WZA6.2), mOR31-4 (NP_667290.2), mOR33-1 (GenBank AAL60676.1), mOR103-15 (NP_035113.1), mOR106-13 (NP_001011738.1), mOR171-2 (NP_997547.1), mOR174-4 (GenBank BAB59038.1), mOR174-9 (NP_473431.1), mOR175-1 (SwissProt Q9QY00.1), mOR276-1 (GenBank AAL60877.1), and Olfr226 (SwissProt P23270.2). The rho1D4 epitope (TETSQVAPA) preceeded by a GSSG linker was added to the C-terminus of each receptor to facilitate purification and western blot detection. The codons for each receptor were optimized for E. coli expression. The genes were commercially synthesized by GeneArt (Germany) and subcloned into the pIVex2.3d vector (Roche Diagnostics Corp.). The final constructs were verified by DNA sequencing (MIT Biopolymers Lab, Cambridge, MA).

### Cell-Free Olfactory Receptor Expression

*E. coli* based cell-free expression kits were used to synthesize the olfactory receptors according to the manufacturer's instructions (Invitrogen, K9900-97 and Qiagen). To compensate for the lack of a natural membrane, surfactants were added directly to the reactions. Experimental concentrations of the peptides are shown in Table S1. Brij-35 was used at a final concentration of 0.2% w/v. A final reaction volume of 50 µl was used for all screens. Final reaction volumes of 0.5–1.0 ml were used to produce protein that was purified for secondary structure and binding analyses.

### Olfactory Receptor Detection and Purity Analysis

Western blots and silver stains were used to detect the proteins and analyze their purity. Samples were prepared and loaded in Novex 10% Bis-Tris SDS-PAGE gels (Invitrogen) according to the manufacturer's protocol, with the exception that the samples were incubated at room temperature prior to loading as boiling causes membrane protein aggregation. For blotting, the gel-resolved samples were transferred to a nitrocellulose membrane, blocked in milk (5% w/v non-fat dried milk in TBST) for 1 hour, and incubated with a rho1D4 primary antibody (1∶3000 in TBST, 1 hour at room temperature, or overnight at 4°C). The olfactory receptors were then detected with a goat anti-mouse HRP-conjugated secondary antibody (Pierce, Rockford, IL) (1∶5000 in TBST, 1 hour, room temperature) and visualized using the ECL-Plus Kit (GE Healthcare). The SilverXpress kit (Invitrogen, LC6100) was used according to the manufacturer's instructions to perform total protein stains of the samples. The Full Range Rainbow ladder (GE Healthcare, Waukesha, WI) was used for western blots, and the Benchmark Protein Ladder (Invitrogen, 10747-012) was used for silver staining. All images were captured using a Fluor Chem gel documentaion system (Alpha Innotech, San Leandro, CA).

### Peptide Detergent Screening

Five pairs of peptide detergents were used in the cell-free production of olfactory receptors. As a control, the traditional detergent Brij-35 was also used. The detergents were tested at concentrations above their determined or estimated critical micelle concentrations (CMCs). Cell-free reactions were performed according to the manufacturer's instructions. Briefly, plasmid DNA, detergent, and the reaction reagents were incubated at 30°C and 300 rpm for 30 minutes. A feed buffer was added, and the reaction was incubated for an additional 90 minutes. The samples were then centrifuged at 10,000 rpm for 5 minutes. The supernatant containing the solubilized protein was removed, and the pellet was resuspended in an equivalent volume of PBS. The relative quantities of solubilized and precipitated protein were determined with a western or dot blot. ImageJ (http://rsb.info.nih.gov/ij/) was used to perform all densitometry analyses.

### Immunoaffinity Purification

CNBr-activated Sepharose 4B beads (GE Healthcare) chemically linked to the rho1D4 monoclonal antibody (Cell Essentials, Boston, MA) were used for immunoaffinity purification. Solubilized protein from the cell-free reactions was mixed with the bead slurry (binding capacity 0.7 mg/ml) and rotated overnight at 4°C to capture the synthesized protein. The beads were then washed with wash buffer (PBS + 0.2% FC-14 w/v) until spectrophotometer readings indicated that all excess protein had been removed (<0.01 mg/ml). The captured ORs were eluted with elution buffer (PBS + 0.2% FC-14 + 800 µM elution peptide). The elution peptide Ac-TETSQVAPA-CONH_2_ was synthesized by CPC Scientific Inc., CA. Elutions were performed until spectrophotometer readings indicated that no more protein was present (<0.01 mg/ml). The protein was concentrated using 30 kDa or 50 kDa MWCO filter columns (Millipore, Billerica MA), and was washed with >20 volumes of wash buffer to remove residual elution peptide. All concentrations were measured using the NanoDrop 1000 spectrophotometer (Thermo Scientific). For some samples, the concentration was also measured with a total protein stain by comparing the intensity of the OR band to the intensity of a BSA band of known concentration. The beads were pelleted by centrifugation at 1,400×g for one minute between each wash and elution.

### Circular Dichroism Analyses

Spectra were recorded on a CD spectrometer (Aviv Associates, model 410) at 15°C over the wavelength range of 195–250 nm with a step size of 1 nm and an averaging time of 4 seconds. Spectra for purified ORs were blanked to wash buffer. A 111-QS quartz sample cell with a path length of 1 mm (Hellma, USA) was used. 300 ul of protein sample was used for each experiment. The spectra were smoothed using an averaging filter with a span of 5.

### Microscale Thermophoresis Binding Analyses

Thermophoresis is the directed movement of molecules along a temperature gradient. A spatial temperature difference ΔT leads to a depletion of molecules in the region of elevated temperature, quantified by the Soret coefficient S_T_ and the local molecular concentration c:
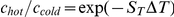
Under constant buffer conditions, thermophoresis mainly probes the solvation entropy of molecules, and thus depends on their conformation. The thermophoresis of a receptor alone typically differs significantly from that of a receptor-ligand complex due to binding induced changes in solvation energy or conformation. This difference in a molecule's thermophoresis is used to quantify the binding in titration experiments by plotting the measured microscale thermophoresis signal against a varying ligand concentration.

Thermophoresis was used to measure the binding interactions between purified receptors and their ligands using a setup similar to that previously described [24]. To eliminate artifacts caused by labeling or modifying proteins, the fluorescence of native olfactory receptor tryptophans was used to monitor the local receptor concentration. For each tested olfactory receptor, a titration series with constant olfactory receptor concentration and varying ligand concentrations was prepared in a final solution of 10% DMSO and 0.2% FC-14 in PBS. Potential autofluorescence of each ligand was checked: no fluorescence signal was detected from the ligands in the tryptophan fluorescence channel. The final receptor concentration was 2 µM, except for mOR103-15 in Ac-V_3_D-OH, which was 1 µM. Approximately 1.5 µL of each sample was loaded in a fused silica capillary (Polymicro Technologies, Phoenix, USA) with an inner diameter of 300 µm. An infrared laser diode was used to create a 0.12 K/µm temperature gradient inside the capillaries (Furukawa FOL1405-RTV-617-1480, wavelength λ = 1480 nm, 320 mW maximum power, AMS Technologies AG, Munich Germany). Tryptophan fluorescence was excited with a UV-LED (285 nm). Emission was measured at 350±20 nm with a 40× SUPRASIL synthetic quartz substrate microscope objective, numerical aperture 0.8 (Partec, Goerlitz, Germany). The local receptor concentration in response to the temperature gradient was detected with a photon counter PMT P10PC (Electron Tubes Inc, Rockaway, NJ, USA). All measurements were performed at room temperature. Fluorescence filters for tryptophan (F36-300) were purchased from AHF-Analysentechnik (Tübingen, Germany). The Hill equation (n = 2) was fit to the data to determine the EC50 value for each sample; the EC50 value is the concentration at which half of the OR sample is bound to its ligand.

## Supporting Information

Table S1 Surfactant peptide properties and experimental concentrations.
(DOC)

## References

[1] Garavito RM, Picot D, Loll PJ (1996). Strategies for crystallizing membrane proteins.. J Bioenerg Biomembr.

[2] Prive CG (2007). Detergents for the stabilization and crystallization of membrane proteins.. Methods.

[3] Schafmeister CE, Miercke LJ, Stroud RM (1993). Structure at 2.5 Å of a designed peptide that maintains solubility of membrane proteins.. Science.

[4] McGregor CL, Chen L, Pomroy NC, Hwang P, Go S (2003). Lipopeptide detergents designed for the structural study of membrane proteins.. Nat Biotechnol.

[5] Popot JL, Berry EA, Charvolin D, Creuzenet C, Ebel C (2003). Amphipols: Polymeric surfactants for membrane biology research.. Cell Mol Life Sci.

[6] Thiesen MJ, Potocky TB, McQuade DT, Gellman SH, Chiu ML (2005). Crystallization of bacteriorhodopsin solubilized by a tripod amphiphile.. Biochim Biophys Acta.

[7] Tribet C, Audebert R, Popot JL (1996). Amphipols: Polymers that keep membrane proteins soluble in aqueous solutions.. Proc Natl Acad Sci USA.

[8] Yu SM, McQuade DT, Quinn MA, Hackenberger CPR, Krebs MP (2000). An improved tripod amphiphile for membrane protein solubilization.. Protein Science.

[9] Vauthey S, Santoso S, Gong H, Watson N, Zhang S (2002). Molecular self-assembly of surfactant-like peptides to form nanotubes and nanovesicles.. Proc Natl Acad Sci USA.

[10] Santoso S, Hwang W, Hartman H, Zhang S (2002). Self-assembly of surfactant-like peptides with variable glycine tails to form nanotubes and nanovesicles.. Nano Letters.

[11] von Maltzahn G, Vauthey S, Santoso S, Zhang S (2003). Positively charged surfactant-like peptides self-assemble into nanostructures.. Langmuir.

[12] Nagai A, Nagai Y, Qu H, Zhang S (2007). Self-assembling behaviors of lipid-like peptides A_6_D and A_6_K.. J Nanoscience & Nanotechnology.

[13] Khoe U, Yanlian Y, Zhang S (2009). Self-assembly of nano-donut structure from cone-shaped designer lipid-like peptide surfactant.. Langmuir.

[14] Yaghmur A, Laggner P, Zhang S, Rappolt M (2007). Tuning curvature and stability of monoolein bilayers by designer lipid-like peptide surfactants.. PLoS ONE.

[15] Yeh JI, Du S, Tordajada A, Paulo J, Zhang S (2005). Peptergent: peptide detergents that improve stability and functionality of a membrane protein glycerol-3-phosphate dehydrogenase.. Biochemistry.

[16] Kiley P, Zhao X, Bruce BD, Baldo M, Zhang S (2005). Self-assembling peptide detergents stabilize isolated photosystem I on a dry surface for an extended time.. PloS Biology.

[17] Matsumoto K, Koutsopoulos S, Vaughn M, Bruce BD, Zhang S (2009). Designer lipid-like peptide surfactants stabilize functional Photosystem I membrane complex in solution.. J Phys Chem.

[18] Zhao X, Nagai Y, Revees P, Kiley P, Khorana HG, Zhang S (2006). Designer lipid-like peptides significantly stabilize G-protein coupled receptor bovine rhodopsin.. Proc Natl Acad Sci USA.

[19] Wang X, Corin K, Baaske P, Wienken CJ, Jerabek-Willemsen M (2011). Peptide Surfactants for Cell-Free Production of Functional G Protein-Coupled Receptors.. Proc Natl Acad Sci USA.

[20] Corin K, Baaske P, Ravel DB, Song J, Brown E (2011). A robust and rapid method of producing soluble, stable, and functional G-protein coupled receptors.. PLoS One.

[21] Cook BL, Ernberg KE, Chung H, Zhang S (2008). Study of a Synthetic Human Olfactory Receptor 17-4: Expression and Purification from an Inducible Mammalian Cell Line.. PLoS One.

[22] Cook BL, Steuerwald D, Kaiser L, Graveland-Bikker J, Vanberghem M (2009). Large scale production and study of a synthetic G-protein coupled receptor: Human olfactory receptor 17-4.. Proc Natl Acad Sci USA.

[23] Kaiser L, Graveland-Bikker J, Steuerwald D, Vanberghem M, Herlihy K, Zhang S (2008). Efficient cell-free production of olfactory receptors: Detergent optimization, structure, and ligand binding analyses.. Proc Natl Acad Sci USA.

[24] Baaske P, Wienken CJ, Reineck P, Duhr S, Braun D (2010). Optical thermophoresis for quantifying the buffer dependence of aptamer binding.. Angew Chem Int Ed.

[25] Duhr S, Braun D (2006). Why molecules move along a temperature gradient.. Proc Natl Acad Sci USA.

[26] Wienken CJ, Baaske P, Rothbauer U, Braun D, Duhr S (2010). Protein Binding Assays in Biological Liquids using Microscale Thermophoresis.. Nat Comm.

[27] Spirin AS, Baranov VI, Ryabova LA, Ovodov SY, Alakhov YBA (1988). Continuous cell-free translation system capable of producing polypeptides in high yield.. Science.

[28] Yokoyama S (2003). Protein expression systems for structural genomics and proteomics.. Curr Opin Chem Biol.

[29] Endo Y, Sawasaki T (2006). Cell-free expression systems for eukaryotic protein production.. Curr Opin Biotechnol.

[30] Ishihara G, Goto M, Saeki M, Ito K, Hori T (2005). Expression of G protein coupled receptors in a cell-free translational system using detergents and thioredoxin-fusion vectors.. Protein Expr Purif.

[31] Klammt C, Schwarz D, Fendler K, Haase W, Dotsch V, Berhard F (2005). Evaluation of detergents for the soluble expression of alpha-helical and beta-barrel-type integral membrane proteins by a preparative scale individual cell-free expression system.. FEBS J.

[32] Savage DF, Anderson CL, Robles-Colmenares Y, Newby ZE, Stroud RM (2007). Cell-free complements in vivo expression of the E. coli membrane proteome.. Protein Sci.

[33] Klammt C, Schwarz D, Eifler N, Engel A, Piehler J (2007). Cell-free Production of G Protein-Coupled Receptors for Functional and Structural Studies.. J Structural Biology.

