# Supplementary material for: Designer Lipid-Like Peptides: A Class of Detergents for Studying Functional Olfactory Receptors Using Commercial Cell-Free Systems
Source: PLoS One. 2011 Nov 23;6(11):e25067. doi: 10.1371/journal.pone.0025067 (PMC3223156; doi:10.1371/journal.pone.0025067)
Supplement: Table S1 — Surfactant peptide properties and experimental concentrations. (DOC) [file pone.0025067.s001.doc]

**Table S1.** Surfactant peptide properties and experimental concentrations

| Peptide (1-letter code) | Molecular weight (kDa) | Net charge at pH 7.0 | Experimental Concentration^2^ | CAC in water (mM)^1^ |
| --- | --- | --- | --- | --- |
| Ac-VVVD | 472.5 | -2 | 0.625mM^2^ | 2.3 |
| Ac-VVVK-CONH2 | 484.6 | +1 | 2.5mM | 1.6 |
| Ac-IIID | 514.6 | -2 | 0.625mM^2^ | 1.0-1.2 |
| Ac-IIIK-CONH2 | 526.7 | +1 | 0.625mM | 0.4-0.5 |
| Ac-LLLD | 514.6 | -2 | 2.5mM | 1.2 |
| Ac-LLLK-CONH2 | 526.7 | +1 | 2.5mM | 1.2 |
| Ac-AAAAAAD | 601.6 | -2 | 1.7 mM | 0.3 |
| Ac-AAAAAAK-CONH2 | 613.7 | +1 | 1.6mM | 0.2 |
| DAAAAAA-CONH2 | 558.6 | 0 | 2.5mM | 0.2 |
| KAAAAAA-CONH2 | 571.7 | +2 | 0.5mM | 0.3 |
